# A randomized clinical trial demonstrating cell type specific effects of hyperlipidemia and hyperinsulinemia on pituitary function

**DOI:** 10.1371/journal.pone.0268323

**Published:** 2022-05-11

**Authors:** Rosemary McDonald, Katherine Kuhn, Thy B. Nguyen, Andrew Tannous, Irene Schauer, Nanette Santoro, Andrew P. Bradford

**Affiliations:** 1 Department of Obstetrics and Gynecology, University of Colorado School of Medicine, Aurora, CO, United States of America; 2 Department of Medicine, Division of Endocrinology, Metabolism & Diabetes, University of Colorado School of Medicine, Aurora, CO, United States of America; 3 Endocrinology Section, Rocky Mountain Regional Veterans Affairs Medical Center, Aurora, CO, United States of America; Weill Cornell Medical College Qatar, QATAR

## Abstract

**Introduction:**

Obesity is characterized by elevated lipids, insulin resistance and relative hypogonadotropic hypogonadism, reducing fertility and increasing risk of pregnancy complications and birth defects. We termed this phenotype ‘Reprometabolic Syndrome’ and showed that it can be recapitulated by acute infusions of lipid/insulin into healthy, normal weight, eumenorrheic women. Herein, we examined the broader impact of hyperlipidemia and euglycemic hyperinsulinemia on anterior pituitary trophic hormones and their targets.

**Methods:**

Serum FSH, LH, TSH, growth hormone (GH), prolactin (PRL), thyroid hormones (free T4, total T3), cortisol, IGF-1, adiponectin, leptin and creatinine were measured in a secondary analysis of an interventional crossover study of 12 normal weight cycling women who underwent saline and heparin (control) infusion, or a euglycemic insulin infusion with heparin and Intralipid^®^ (lipid/insulin), between days 2–5 in sequential menstrual cycles.

**Results:**

In contrast to the decrease in gonadotropins, FSH and LH, infusion of lipid/insulin had no significant effects on other trophic hormones; TSH, PRL or GH. Thyroid hormones (fT4 and total T3), cortisol, IGF-1, adiponectin and creatinine also did not differ between saline or lipid/insulin infusion conditions. Leptin increased in response to lipid/insulin (p<0.02).

**Conclusion:**

Acute hyperlipidemia and hyperinsulinemia exerted differential, cell type specific effects on the hypothalamic-pituitary-gonadal, adrenal and thyroid axes. Elucidation of mechanisms underlying the selective modulation of pituitary trophic hormones, in response to changes in diet and metabolism, may facilitate therapeutic intervention in obesity-related neuroendocrine and reproductive dysfunction.

## Introduction

Obesity has a profound impact on reproductive function, reducing fertility and increasing the risk of pregnancy complications and birth defects [1, 2]. Obesity in women is also associated with increased circulating free fatty acids, insulin resistance, and is characterized by decreased basal and GnRH-stimulated FSH and LH secretion from the pituitary [3, 4]. There is evidence to suggest that obesity also dysregulates non-reproductive hypothalamic-pituitary (HP) axes [5, 6]. However, the mechanisms underlying disruption of these neuroendocrine pathways is controversial, poorly understood, and specific mediators remain to be identified.

We have previously shown that acute infusions of lipid and insulin into normal weight women recapitulates the obesity phenotype of decreased basal and GnRH-stimulated LH and FSH secretion, which we have termed ‘Reprometabolic Syndrome’ [7, 8]. Gonadotrophs appear to be negatively impacted, with respect to trophic hormone secretion, by increased levels of circulating free fatty acids and insulin; however, the impact of experimental hyperinsulinemia and hyperlipidemia on other pituitary cell types and the underlying mechanisms remain to be elucidated.

The goals of this study were to investigate the effects of acute infusions of lipid and insulin, previously shown to suppress gonadotropin levels [7, 8] on other anterior pituitary cell types, trophic hormones and their regulators.

## Materials and methods

### Participants

This study was performed as a secondary analysis of a subset of samples, from an ongoing parent study, investigating the effect of acute infusions of lipid and insulin on the reproductive hormonal output of normal weight (BMI 18.5–24.9 kg/m^2^), regularly cycling women [8]. Eligible women were of reproductive age (mean age 30.6 ±4.9) and normal weight (mean BMI 21.4 ±1.37 kg/m^2^), with a history of regular menstrual cycles every 25–35 days, no more than moderate exercise (<4 hours per week), and a normal screening TSH and prolactin. One participant was excluded from the analysis of serum TSH, as her levels on study exceeded the range of accepted values for euthyroidism [9, 10], reaching maximums of almost 7μIU/ml during the saline infusion, although her TSH was normal at screening. We did not conduct ovarian ultrasounds; however, none of the women exhibited clinical signs of polycystic ovary syndrome (PCOS), with normal HbA1c, fasting glucose, no hirsutism, regular menses, and no evidence of insulin resistance [8].

### Statement of ethics

The protocol (CRV005-1) was approved by the Colorado Multiple Institutional Review Board (COMIRB), registered at ClinicalTrials.gov (NCT02653092), and was performed in accordance with the ethical standards as laid in the 1964 Declaration of Helsinki and its later amendments or comparable ethical standards. Informed written consent was obtained from all individual participants included in the study. The authors affirm that human research participants provided informed consent for publication of data. No identifying information or images are included in the manuscript.

### Study design

**Fig 1:**
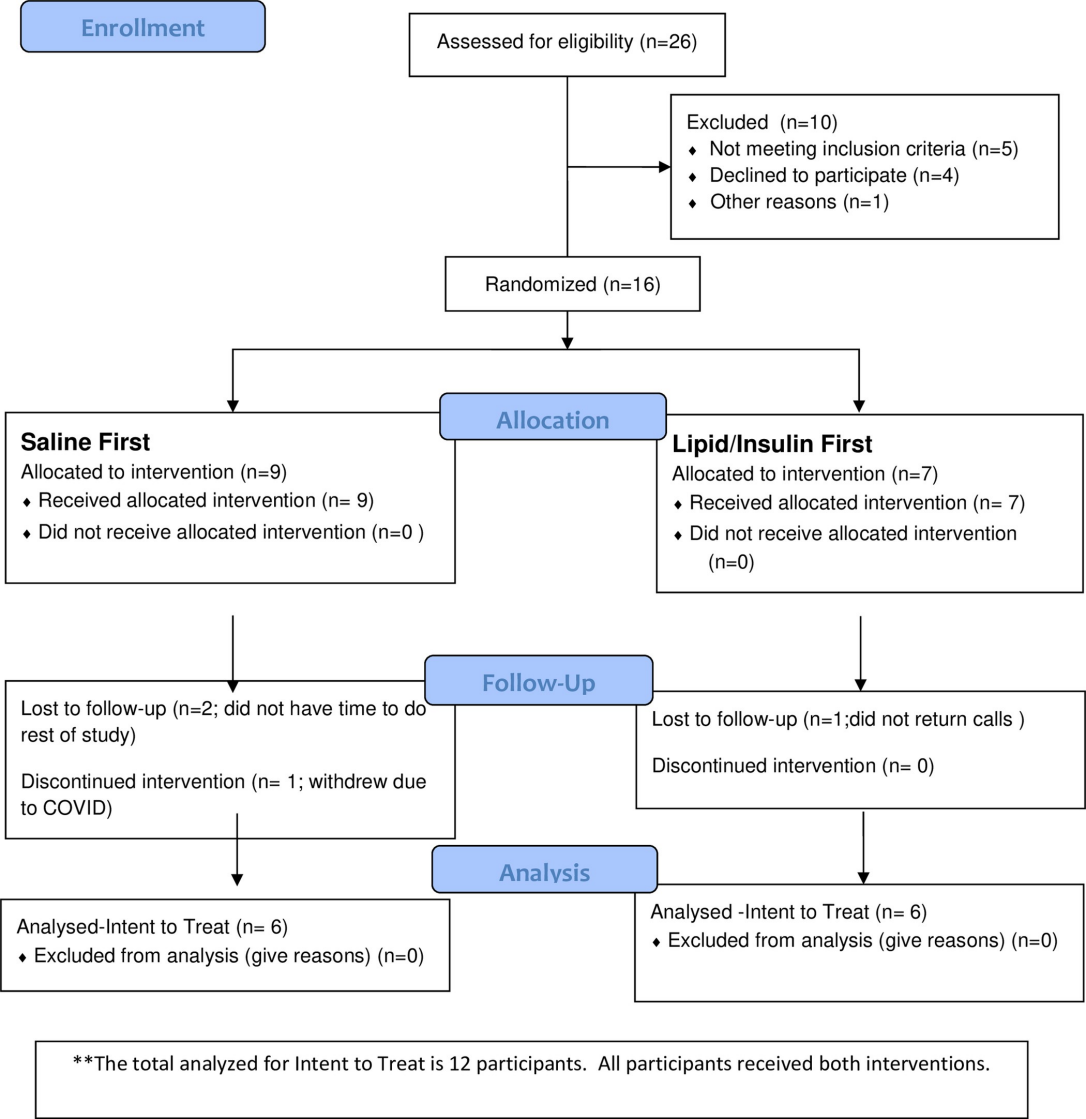
Flow diagram showing process for participant screening, enrollment and randomization for study visit infusions. The total participants analyzed was 12.

Secondary analysis of serum samples from 12 women who participated in both arms of the ongoing parent study at the University of Colorado School of Medicine, between February 2016 and March 2020, was performed (Fig 1). Each woman underwent two 6-hour visits; a euglycemic lipid/heparin plus insulin infusion, or a saline/heparin infusion (control). Both visits occurred in random order, determined by a research randomizer program (www.randomizer.org), in sequential menstrual cycles, during the follicular phase (day 2–5 of cycle). 6 participant’s infusions were carried out in consecutive cycles, 5 completed both infusions within 3 months, and 1 within 7 months. During each visit, blood samples were taken at 10-minute intervals, with a weight-based, physiologic dose of GnRH given at 240 minutes (75ng/kg).

### Insulin, dextrose and lipid/heparin infusion

Heparin (0.4U/Kg/min) was co-administered with lipid infusion to enhance liberation of free fatty acids as described previously [7]. Partial thromboplastin time (PTT) was checked prior to and during infusions and the rate was adjusted to maintain PTT in the protocol approved range (<120 seconds). Control (saline/heparin) or lipid/heparin (Intralipid^®^, 20% emulsion, 45ml/h) plus insulin (20-40mU/m^2^/min) infusions occurred over separate 6-hour visits each beginning at 07:00h. Steady state serum levels of insulin and fatty acids were achieved approximately 2–3 hours into the infusion [7, 8]. Glucose was checked every 5 minutes during the 6 hour lipid/insulin infusion, and 20% dextrose was co-infused through a separate IV line to maintain euglycemia throughout the frequent sampling study. Glucose levels (mean ± SEM) at equilibrium state (2-6h) were 83.9 ± 1.4 mg/dL and 85.9 ± 1.5 mg/dL for saline and lipid/insulin infusions, respectively. Given the rapid metabolism of insulin and lipids and the minimum of one month washout between infusions, it is extremely unlikely that any carry over effect would be observed. However, to address this possibility, we compared the average baseline levels of insulin and free fatty acids, immediately prior to the infusions. Insulin levels (mean ± S.D.) were 3.11 ± 1.6 μIU/ml and 2.15 ± 2.11 μIU/ml for the saline and lipid/insulin infusions, respectively. Baseline serum free fatty acids (mean ± S.D.) were 632 ± 268 μEq/L for saline and 537 ± 210 μEq/L for lipid/insulin infusions. Values between infusions were not significantly different (p = 0.8 and 0.97, respectively) indicating no carry over effects in this crossover study.

### Hormone assays

Serum FSH, LH, TSH, PRL, free thyroxine (fT_4_) and tri-iodothyronine (T_3_), and cortisol were measured using Siemens (Malvern, PA) ADVIA Centaur^®^ XP platform as described previously [7, 8]. Inter-assay and intra-assay coefficients of variation (CV) were 4.4% and 5.1% for FSH, and 4.0% and 2.9% for LH, respectively. Due to limited sample volumes, TSH samples were pooled to be measured approximately every 30 min by combining equal aliquots of three 10-minute time points. Inter- and intra-assay CVs were 3.9% and 3.1%, respectively. PRL, fT_4_, and T_3_ samples were similarly pooled to measure at the 0, 30, 160, and 360 minute time points. PRL inter- and intra-assay CVS were 5.0% and 3.5%, respectively. Inter- and intra-assay CVs were 4.1% and 2.2% for fT_4_, and 6.4% and 4.4% for T_3_, respectively. Growth Hormone (GH) and Insulin-like growth factor 1 (IGF-1) were determined by immunoassay (Beckman Coulter) in the University of Colorado Clinical Laboratory. Inter- and intra-assay CVs were 4.8% and 3.4% for GH, and <12% and <10% for IGF-1, respectively. Serum creatinine was measured using a colorimetric assay kit (Cayman Chemical, Ann Arbor, Michigan) and was measured hourly in pooled samples. Inter and intra-assay CVS were 4.6% and 6.4%, respectively. Leptin and Adiponectin (high molecular weight; RRID:AB_2892778), were measured by ELISA (ALPCO, Salem, NH). Inter- and intra-assay CVs were 5.8% and 3.7% for Leptin, and 4.0% and 2.7% for Adiponectin, respectively.

### Statistical analysis

For each hormone and creatinine, after assessing data for normality, repeated measures analysis of variance was used to determine differences between the saline and lipid/insulin infusions. In addition, paired t-tests were performed to assess for differences at each time point. Leptin, adiponectin and IGF-were analyzed by paired t test. Data were analyzed using STATA version 10.2 (College Station, TX) and graphed using GraphPad Prism 8.3.0 (San Diego, California).

## Results

### Lipid/Insulin infusion decreases LH and FSH levels

**Fig 2:**
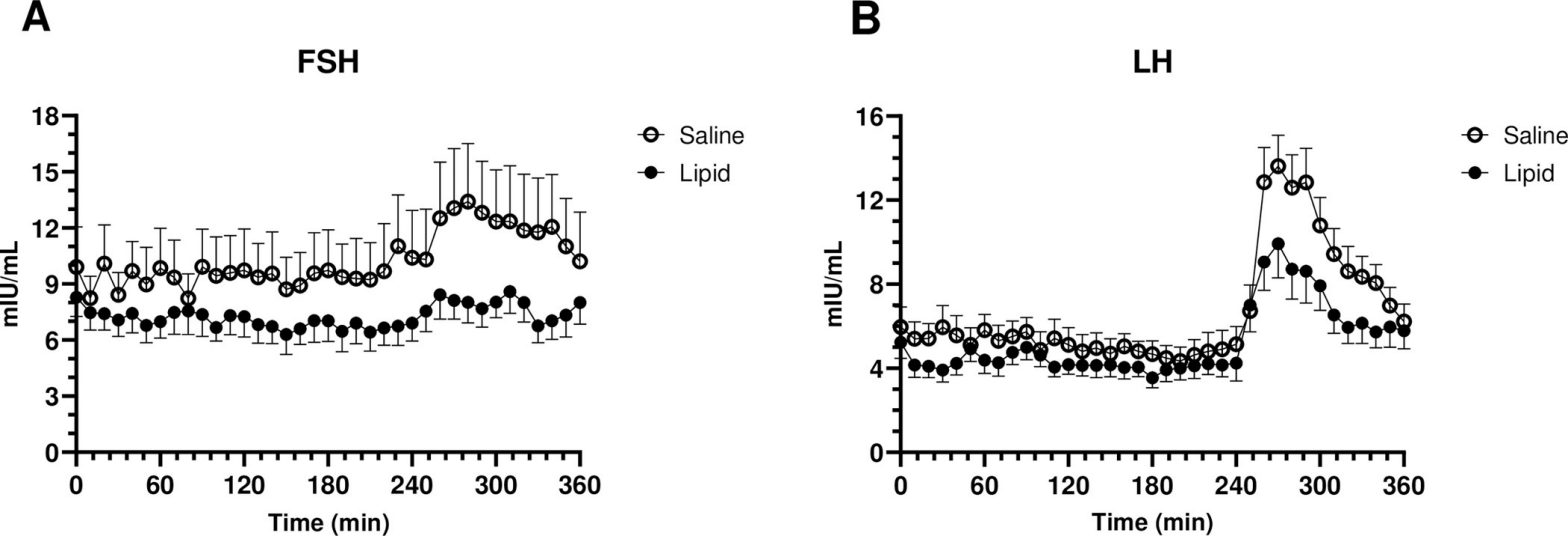
Basal and stimulated gonadotropin levels are decreased in response to acute insulin/lipid infusion. FSH (A) and LH (B) were measured at 10-minute intervals as described [7, 8]. GnRH (75ng/Kg) was administered at 240 min. Values are means ± SEM; n = 12.

We have previously demonstrated that acute hyperlipidemia and hyperinsulinemia, mimicking levels observed in obesity and metabolic syndrome, decreases both basal and GnRH stimulated FSH and LH in normal weight women [7, 8]. This suppression of gonadotropins in response to infusion of lipid/insulin, for the participants studied in this secondary, paired analysis, is illustrated in Fig 2. Mean baseline (0–230 min) FSH and LH values during lipid/insulin infusions were significantly lower than those observed in the corresponding control saline infusion (p<0.05). Similarly, transverse mean GnRH stimulated FSH and LH levels (240–360 min) in the lipid/insulin infusion were reduced compared to saline controls (p<0.02).

### Creatinine levels demonstrate no differential hemodilution

**Fig 3:**
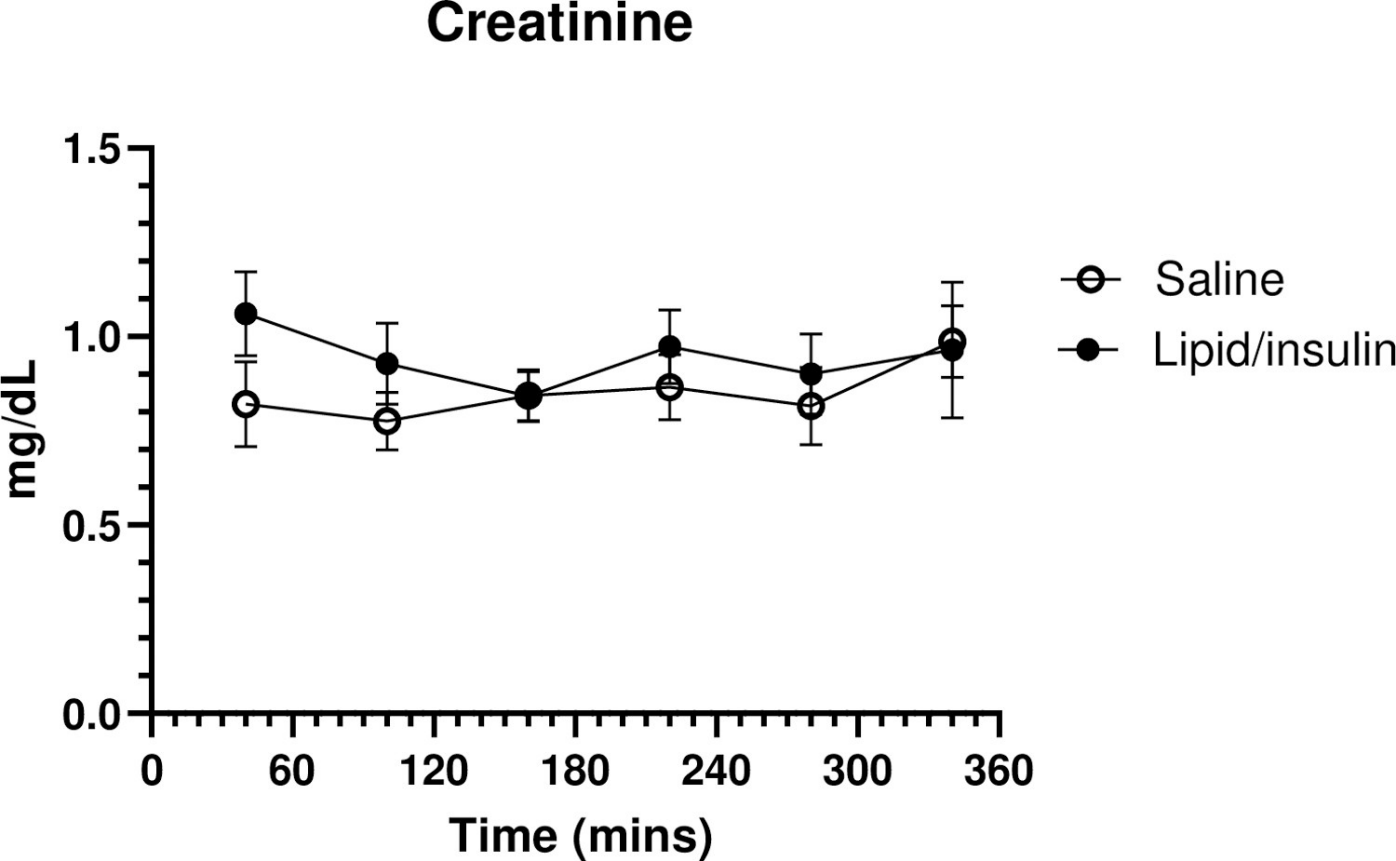
Creatinine levels are unaffected by lipid/insulin, confirming there is no hemodilution occurring. Pooled levels of serum Creatinine were measured, as described in methods, approximately every hour; values are mean ± SEM; n = 12.

In order to maintain euglycemia, variable amounts of glucose were administered during the lipid/insulin infusions, potentially resulting in differential hemodilution. To address this, serum creatinine was chosen as a marker of blood volume, due to its relatively constant rate of production and proven reliability as an indicator of changing hemodilution [11]. Creatinine, levels did not differ between saline and lipid/insulin visits and remained stable throughout both 6-hour infusion protocols (Fig 3).

### Effects of Lipid/insulin infusion on TSH and thyroid hormones

**Fig 4:**
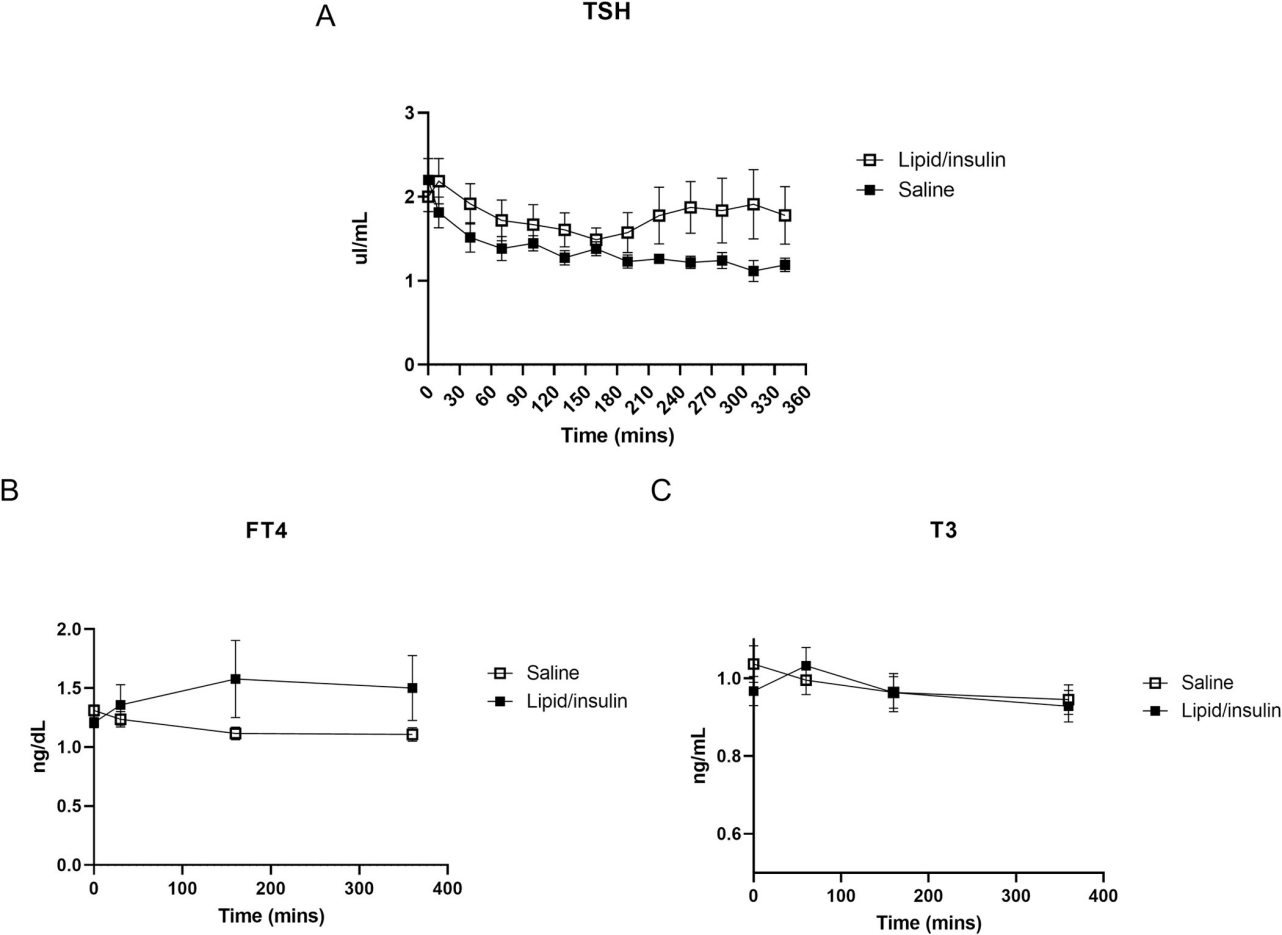
Lipid/insulin infusions may increase relative serum TSH levels but have no effect on thyroid hormones. Hormones were measured as described in methods. (A) Pooled levels of serum TSH were measured every 30 minutes; values are mean ± SEM (n = 11). Pooled levels of (B) serum FT4 and (C) T3 measured at approximately 0, 30, 160, and 360-minutes; values are mean ± SEM (n = 11).

TSH levels initially followed the typical diurnal pattern [12], declining gradually from 0 to 160 minutes. However, a modest increase in TSH was observed in the lipid/insulin-treated participants at later time points (Fig 4A), such that the area under the curve (AUC), reflecting total TSH exposure, between 210 and 360 mins, was greater (mean AUC ± SEM was 180 ± 10.6 and 269 ± 50.4 for saline and lipid/insulin infusions, respectively; p = 0.07). Mean levels of TSH also trended higher in the lipid/insulin infusion compared to saline at later time points (210–360 mins) but did not reach statistical significance (p = 0.052–0.096).

We next investigated whether lipid/insulin infusion affected thyroid hormone levels. Both fT_4_ (Fig 4B) and T_3_ (Fig 4C) were stable over the 6-hour visits, and there were no differences in levels between the saline and lipid/insulin visits.

### Prolactin and Cortisol levels are not affected by lipid/insulin

**Fig 5:**
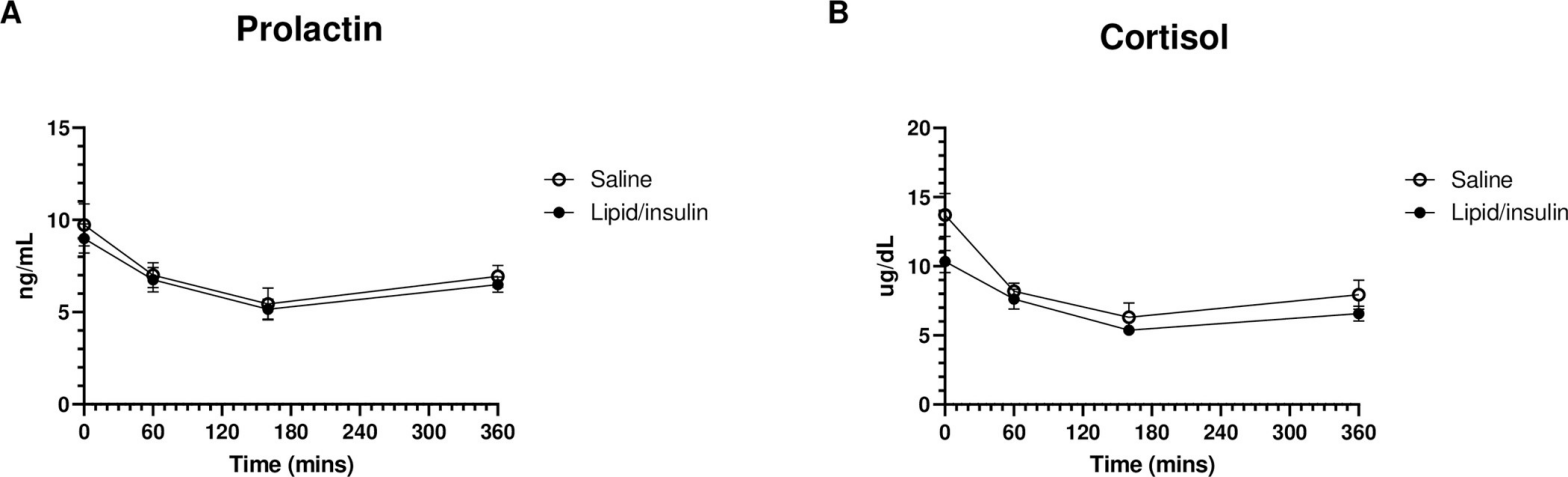
Prolactin and cortisol are unaffected by lipid/insulin infusions. Pooled levels of (A) serum prolactin and (B) cortisol (n = 12) were measured at approximately 0, 60, 160, and 360-minutes, as described in methods; values are mean ± SEM.

To determine the effects on pituitary lactotroph and corticotroph cells, we measured serum levels of prolactin and cortisol. No significant differences in prolactin levels were observed, at any timepoints, between the saline control and the lipid/insulin infusions (Fig 5A). Similarly, serum cortisol did not significantly differ between the saline control and lipid/insulin visits (Fig 5B), and followed the typical diurnal variation seen in cortisol levels, over all of the 6-hour visits, with levels highest in the morning after waking and a subsequent decrease throughout the day [13].

### Effects of lipid/insulin on growth hormone and IGF-1

**Fig 6:**
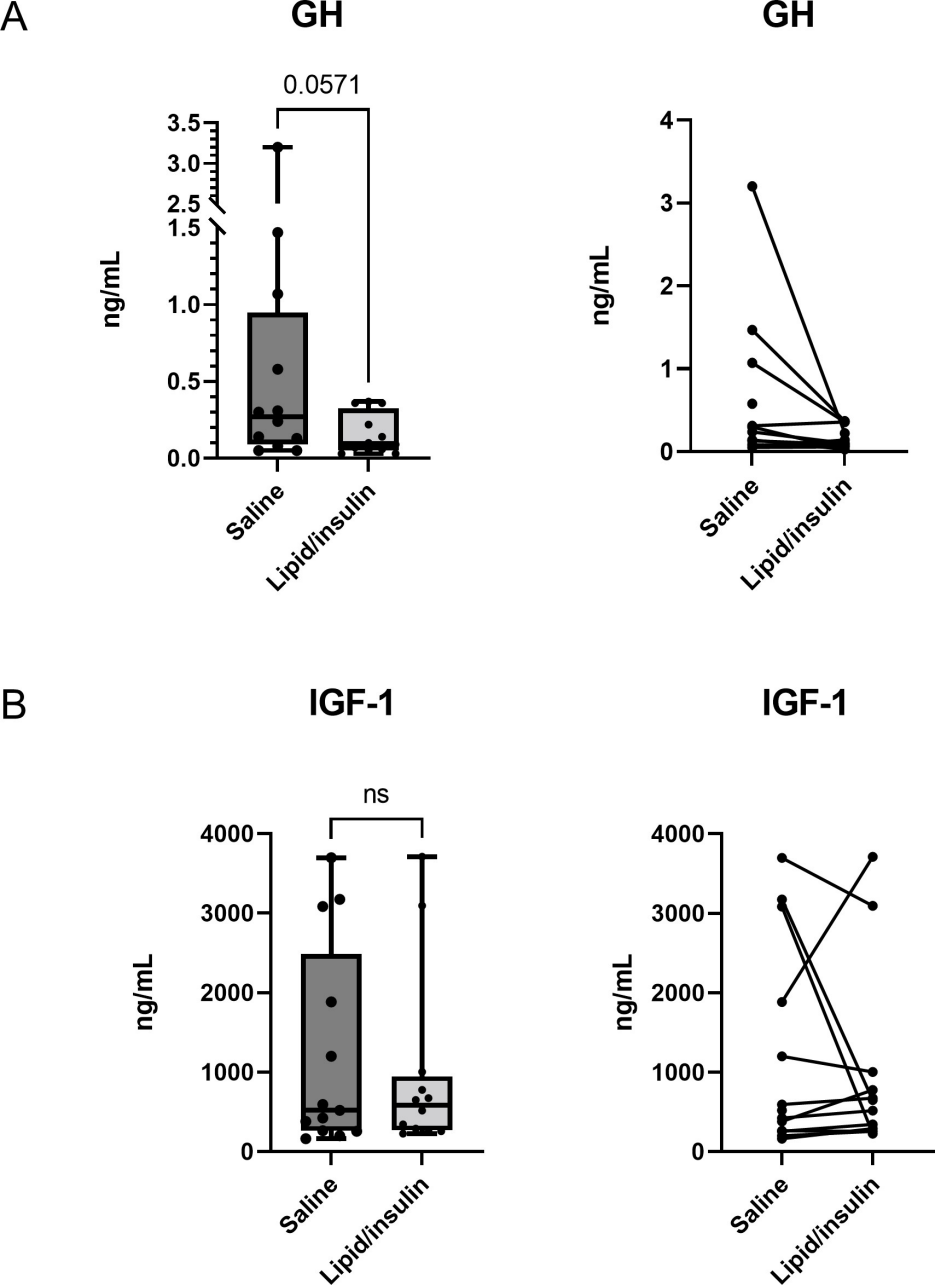
Effect of lipid/insulin infusion on GH and IGF-1. Pooled steady state levels of (A) serum GH and (B) serum IGF-1 were measured as described in methods; boxes encompass 25^th^ -75^th^ percentiles, whiskers are maximum and minimum values, and bar indicates median. Spaghetti plots indicate paired data points for each participant for the saline and lipid/insulin infusions. (n = 12).

To observe whether the GH-IGF-1 axis was affected by lipid/insulin infusions, we measured both hormones in pooled serum (180–230 min) after steady-state was reached (Fig 6A and 6B). We observed a trend toward decreased GH in response to the lipid/insulin infusion (p = 0.057). No significant differences were found in IGF-1 levels between saline and lipid/insulin conditions.

### Lipid/insulin infusion increases leptin levels

**Fig 7:**
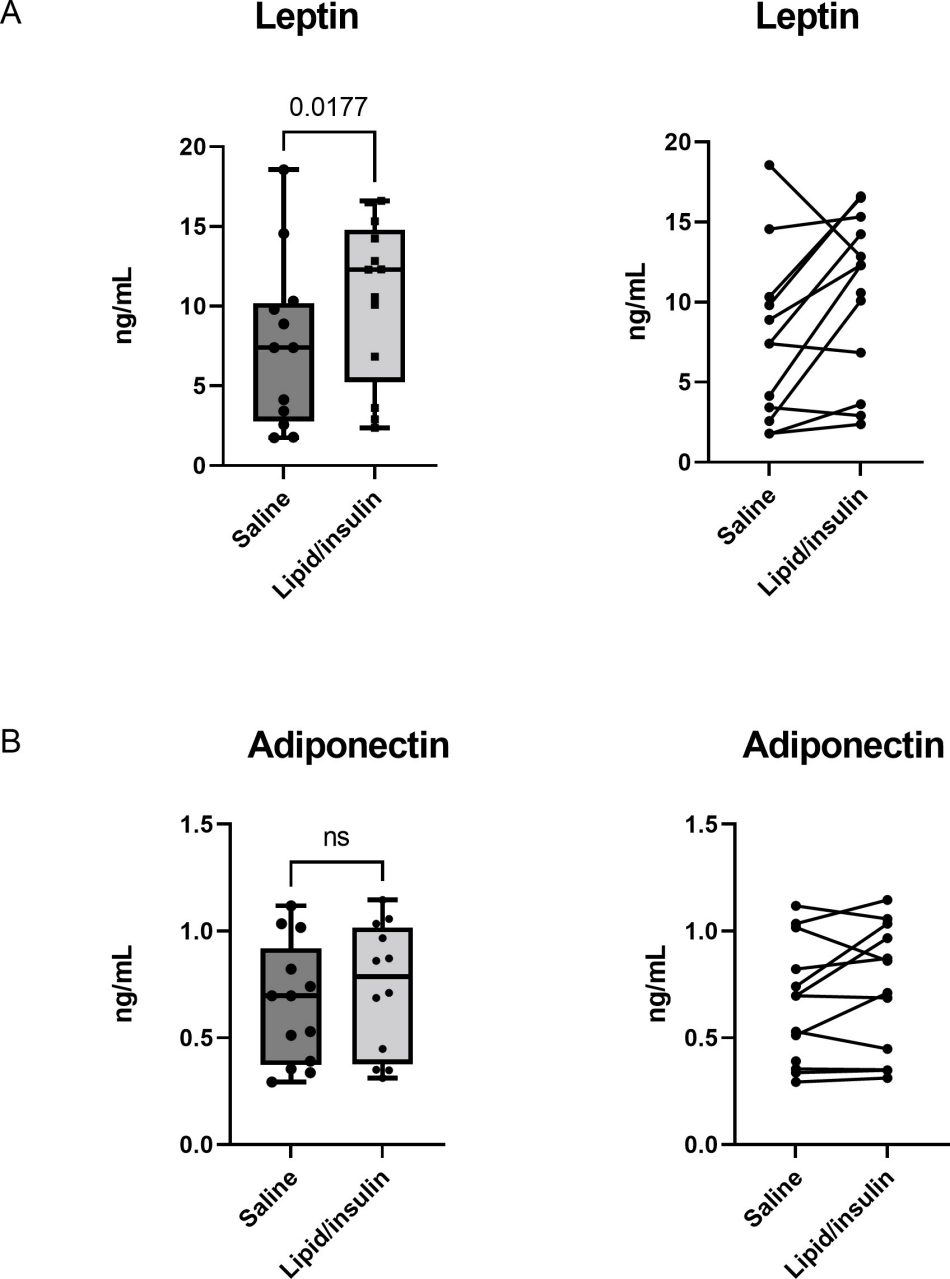
Lipid/insulin infusion increases leptin and has no effect on adiponectin. (A) Pooled levels of serum leptin and (B) adiponectin were measured as described in methods, during saline and lipid/insulin infusion visits; boxes encompass 25^th^ -75^th^ percentiles, whiskers are maximum and minimum values, and bar indicates median. Spaghetti plots indicate paired data points for each participant for the saline and lipid/insulin infusions. (n = 12).

Adipocytokine response to lipid and insulin was investigated as described [14]. Leptin and high molecular weight adiponectin were measured in serum pooled from 12 participants (between 180 and 230 min during steady state levels of insulin and free fatty acids) in both saline and insulin/lipid infusions. Interestingly, there was a slight but statistically significant increase in serum leptin during the lipid/insulin visit compared to the saline control (p<0.02) (Fig 7A). No significant difference in high molecular weight adiponectin levels were observed between the saline and lipid/insulin visits (Fig 7B).

## Discussion

We have previously demonstrated that acute hyperinsulinemia and hyperlipidemia, characteristic of obesity, result in decreased basal and GnRH stimulated levels of the pituitary gonadotropins LH and FSH [7, 8]. This phenomenon, which we refer to as ‘Reprometabolic syndrome’, is recapitulated in the subset of participants used in this secondary analysis. Herein, we have examined the combined effects of acute infusions of lipid and insulin on other anterior pituitary cell types and their hormonal output in normal weight women. We first addressed the potential confounding effect of a euglycemic insulin infusion, which required variable amounts of glucose infusion to maintain euglycemia throughout the study, introducing a possible hemodilution effect on serum hormones. However, the lack of change in serum creatinine, a reliable marker of blood volume [11], strongly implies that there was no hemodilution due to variable infusion volumes. This observation was confirmed by relative levels of multiple hormones (fT_4_, T_3_, PRL, IGF-1, adiponectin, cortisol) that were unchanged over both 6-hour visits, with cortisol being particularly sensitive to hemodilution [15]. Our findings clearly indicate that the observed differential effects on anterior pituitary trophic hormones are not attributable to hemodilution. Additionally, and as expected, administration of a GnRH bolus, to induce FSH and LH, had no significant effect on other hormones measured in this study.

Literature on the regulation of TSH in euthyroid individuals with obesity, is contradictory and there is a paucity of studies investigating the combined effects of both lipid and insulin in patients with normal TSH and thyroid hormone levels [16, 17]. The modest increase in TSH that we observed, in response to insulin and lipid infusion, may reflect differential effects and mechanism of action on gonadotroph versus thyrotroph cells. However, we note that TSH levels remained well within normal levels and both serum fT_4_ and total T_3_ did not demonstrate any variation within-condition over the 6-hour visits, nor did they differ between the saline and lipid/insulin visits, suggesting that the observed TSH response may not be of clinical significance. Gonadotropins, TSH and most other pituitary hormones can be inhibited by cortisol when the hypothalamus-pituitary-adrenal axis is upregulated [18, 19]. However, cortisol, was also unaffected by infusion conditions, suggesting adrenal axis was not impacted by lipid/insulin infusions and did not contribute to the observed difference in TSH levels. TSH has also been shown to also be downregulated by inflammatory cytokines [20] but we previously demonstrated that the acute infusion of insulin and lipid did not significantly impact levels of inflammatory cytokines in this study [14].

Prolactin is known to be modulated by thyroid-releasing hormone (TRH), the direct activator of TSH production and secretion [21]. However, levels of the lactotroph hormone PRL were not impacted by lipid/insulin, implying that any elevation in serum TSH was likely not due to increased hypothalamic TRH and confirming that effects on the pituitary are both complex and cell type specific.

The GH-IGF-1 axis plays extensive roles in the regulation of overall metabolism and regulation of body composition, and is implicated in the both the pathophysiology as well as treatment of obesity [22]. Both spontaneous and stimulated growth hormone secretion are known to be attenuated in obesity [23, 24] and the decreased GH levels observed herein are consistent with previous studies showing that elevated FFA, from lipid/heparin infusion, suppressed GH secretion [25, 26]. Similarly, elevated insulin levels, in the absence of hypoglycemia, are also associated with reduced GH levels [27].

The independent effects of hyperinsulinemia and hyperlipidemia on the pituitary have been studied, with most groups focusing on the roles of insulin, stress, diabetes and hypoglycemia in activating the hypothalamic-pituitary-adrenal axis [28, 29]. Hyperinsulinemia increased CRH, ACTH and corticosterone in rats and a similar increase in ACTH and cortisol was observed in healthy, lean men in response to acute supraphysiologic insulin infusion. However, these studies lacked a control, non-insulin treated group, and we did not observe a rise in cortisol in response to euglycemic hyperinsulinemia and hyperlipidemia compared to controls, in normal weight women.

Interestingly, the adipokine leptin slightly but significantly increased during the lipid/insulin infusions. Starvation lowers thyroid hormone levels, mediated by a decrease in leptin that reduces TRH expression in the hypothalamus and inhibits TSH release from the pituitary [30]. Conversely, leptin treatment blunts the starvation-induced suppression of the gonadal, adrenal and thyroid axes in mice [31]. Leptin administration upregulates hypothalamic TRH expression and stimulates release of TSH from the pituitary [32, 33]. These relationships potentially explain the transient observed increase in TSH associated with lipid/insulin infusion. However, analyses of leptin levels at multiple timepoints, as well as mechanistic studies are required to confirm this notion. Adiponectin was also measured within the same timepoint and showed no significant difference in the lipid/insulin visit compared to the saline control. Adiponectin has been implicated in metabolic syndrome, with reduced levels in insulin resistant states and obesity-related syndromes such as hypertension and type 2 diabetes mellitus [34, 35]. In the thyroid axis, some studies report a correlation of adiponectin and thyroid hormone levels, with increased adiponectin levels in hyperthyroidism, compared to normal or hypothyroid state. However, others found no such associations and no differences in adiponectin levels with respect to thyroid status [32] and the complex relationship between metabolic syndrome, thyroid function and adiponectin remains to be elucidated.

We and others have speculated that the decreased secretion of gonadotropins observed after lipid/insulin infusion may be in part due to deleterious effects of lipid accumulation and induction of endoplasmic reticulum stress, known as lipotoxicity [7, 8, 36, 37]. Since we did not observe a decrease in most other pituitary hormones and noted a transient increase in TSH, our findings argue against a non-specific pituitary lipotoxic affect and imply that gonadotroph, and perhaps somatotroph, cells are selectively targeted under conditions of hyperlipidemia and hyperinsulinemia, characteristic of obesity.

Strengths of our study include the ability to isolate specific factors found in obesity, which may underlie the reproductive and metabolic consequences of increased BMI, by acute infusions into normal weight women, the rigidly controlled experimental conditions, the detailed examination of each participant with frequent blood sampling and the crossover design with each participant serving as their own control. Limitations are the small sample size and the fact that these infusions are acute, spanning 6-hours. Therefore, longer-term investigation including isocaloric, high-fat diet studies will be performed in the future to replicate this paradigm in a more ‘real life’ scenario, reflecting longitudinal diet-induced changes in serum lipids and insulin sensitivity in normal weight women.

## Conclusion

Overall, our results imply that the impact of obesity and Reprometabolic Syndrome on the hypothalamic-pituitary-gonadal axis are complex, cell type specific and not simply related to global pituitary suppression. Further research is needed to elucidate mechanisms underlying the selective modulation of pituitary trophic hormones in response to changes in diet and metabolism, which may provide novel avenues for therapeutic intervention to address the reproductive neuroendocrine dysfunction characteristic of obesity.

## Supporting information

S1 Checklist (DOC)

S1 File (DOCX)
